# The occurrence and development of induced pluripotent stem cells

**DOI:** 10.3389/fgene.2024.1389558

**Published:** 2024-04-18

**Authors:** Yi Chen, Meng Li, Yanqing Wu

**Affiliations:** Department of Cardiology, The Second Affiliated Hospital, Jiangxi Medical College, Nanchang University, Nanchang, China

**Keywords:** induced pluripotent stem cells, iPSCs, OSKM, reprogram, epigenetic barriers

## Abstract

The ectopic expression of four transcription factors, Oct3/4, Sox2, Klf4, and c-Myc (OSKM), known as “Yamanaka factors,” can reprogram or stimulate the production of induced pluripotent stem cells (iPSCs). Although OSKM is still the gold standard, there are multiple ways to reprogram cells into iPSCs. In recent years, significant progress has been made in improving the efficiency of this technology. Ten years after the first report was published, human pluripotent stem cells have gradually been applied in clinical settings, including disease modeling, cell therapy, new drug development, and cell derivation. Here, we provide a review of the discovery of iPSCs and their applications in disease and development.

## Introduction

The process of cell differentiation was once considered irreversible. However, Spemann, 1938 discovered in the early 20th century that egg plasma can reprogram an old blastocyst to form a complete but smaller developing tadpole. In the 1960s, Gurdon et al. discovered somatic cell nuclear transfer (SCNT) (Gurdon et al., 1958; Gurdon, 1962). They transferred the nucleus of a somatic cell to an enucleated egg, which then began to divide. An embryo with the same donor genome as the somatic cell was born, demonstrating that somatic cells carry the same genetic code as fertilized eggs and that activating part of this code enables the cells to be reprogrammed to an early developmental state. This new discovery challenged the “Weismann barrier” theory, which suggested that genetics occurred only through germ cells (eggs and sperm), and in cells in specific states, unnecessary genetic codes were deleted or ultimately inactivated (due to other somatic cells not acting as genetic mediators) (Waddington, 1957). Decades later, the discovery of embryonic stem cells (ESCs) further changed the field of regenerative medicine (Evans and Kaufman, 1981; Martin, 1981; Xu et al., 2002). Later, Yamanaka et al. reported that a new generation of ESC-like cells derived from somatic cells that underwent reprogramming by defined factors (Takahashi and Yamanaka, 2006; Takahashi et al., 2007) exhibited altered transcriptional profiles and chromatin patterns in the initiating somatic cells. These changes lead to their transformation into pluripotent cells, which are called induced pluripotent stem cells (iPSCs). This milestone discovery reignited interest in restoring cell vitality and regenerative development. In 2022, Deng et al.'s team announced the generation of chemically induced pluripotent stem cells (CiPSCs) from human fibroblasts through a stepwise chemical reprogramming strategy (Guan et al., 2022). This method of preparing human CiPSCs has advanced the application of cell reprogramming to a new stage with groundbreaking innovative technology.

In recent years, with the progress of and improvements in medical technology, research on reprogramming has gradually been applied to fields such as regenerative medicine, disease modeling, and drug discovery (Moauro et al., 2022). Many breakthrough results in reprogramming have been achieved both *in vitro* and *in vivo*, including restoring vision and improving the regenerative ability of various organs (Ocampo et al., 2016; Wang et al., 2021; Hishida et al., 2022). However, in certain normal and specific environments, this process can cause potential carcinogenic risks and unexpected loss of tissue function (possibly due to a lack of perfect control over the reprogramming process) (Ito et al., 2022). In this article, we review the emergence, development, and application of reprogramming in diseases.

## Discovery of reprogramming

The development of multicellular organisms involves a series of complex cell division and morphogenesis processes that produce all organs and tissues from a single pluripotent cell or fertilized egg. Our system is composed of hundreds of different cell types. The diversity of cell types endows them with unique genetic information (generated based on different environments and induced genome sequences). The gene regulatory network determines the gene expression program that characterizes each cell type; therefore, cell diversity in a fixed genome requires epigenetic changes. For centuries, people have believed that cell differentiation is a “no-return path” and that a cell cannot be restored to its early progenitor or pluripotent state. However, 17 years ago, Yamanaka’s breakthrough discovery showed that by ectopic expression of four transcription factors, namely, Oct3/4, Sox2, Klf4, and c-Myc (collectively known as OSKM) (Takahashi and Yamanaka, 2006; Takahashi et al., 2007), adult cells could be reprogrammed and transformed into iPSCs, prompting mature combinations to be expressed in a wide range of adult cells, greatly enhancing our understanding of cell identity and suggesting various practical applications of iPSCs.

In the initial experiment, Yamanaka identified 24 candidate genes that were ectopically expressed in the nuclei of mouse fibroblasts, reprogramming the cell to a pluripotent state (mimicking the state of ESCs in both morphology and function). At that time, it was unlikely that iPSCs would require 24 factors, but the number of factors needed for the generation of stem cell-like cells was unknown, and the combination of 24 genes was uncontrollable. Yamanaka et al. used the exclusion method by removing one factor at a time from the 24 genes and then reprogramming the 24 combinations. If the same results were obtained, the gene could be removed from the 24 genes without any concerns. After two rounds of screening, it was confirmed that overexpression of the transcription factors OCT3/4, SOX2, KLF4, and c-MYC in mouse fibroblasts can result in the production of ESC-like cells or iPSCs (Takahashi and Yamanaka, 2006).

Compared to ESCs, the advantage of iPSCs is the abundance of sources, with their differentiation and expression observed in organs such as the intracranial tract, heart, liver, stomach, pancreas, kidney, intestine, and adipose tissue (Chen et al., 2020; Papathanasiou et al., 2021; Chondronasiou et al., 2022a; Du et al., 2022; Guallar, 2023). In recent years, research on the use of pluripotent stem cells to simulate organs, tissues, and other systems in the body has gradually increased. In the more than 10 years since the first report was published, human pluripotent stem cells have become the basis for new cell therapies and drug discovery and have been used in clinical applications such as disease modeling and targeted drugs.

## Mechanisms for regulating reprogramming

In the decades since the first study on reprogramming, many explanations have been proposed for the mechanism of reprogramming. Although there are still some unknowns, the general direction has gradually become clear. Here, we will briefly summarize the results.

**FIGURE 1:**
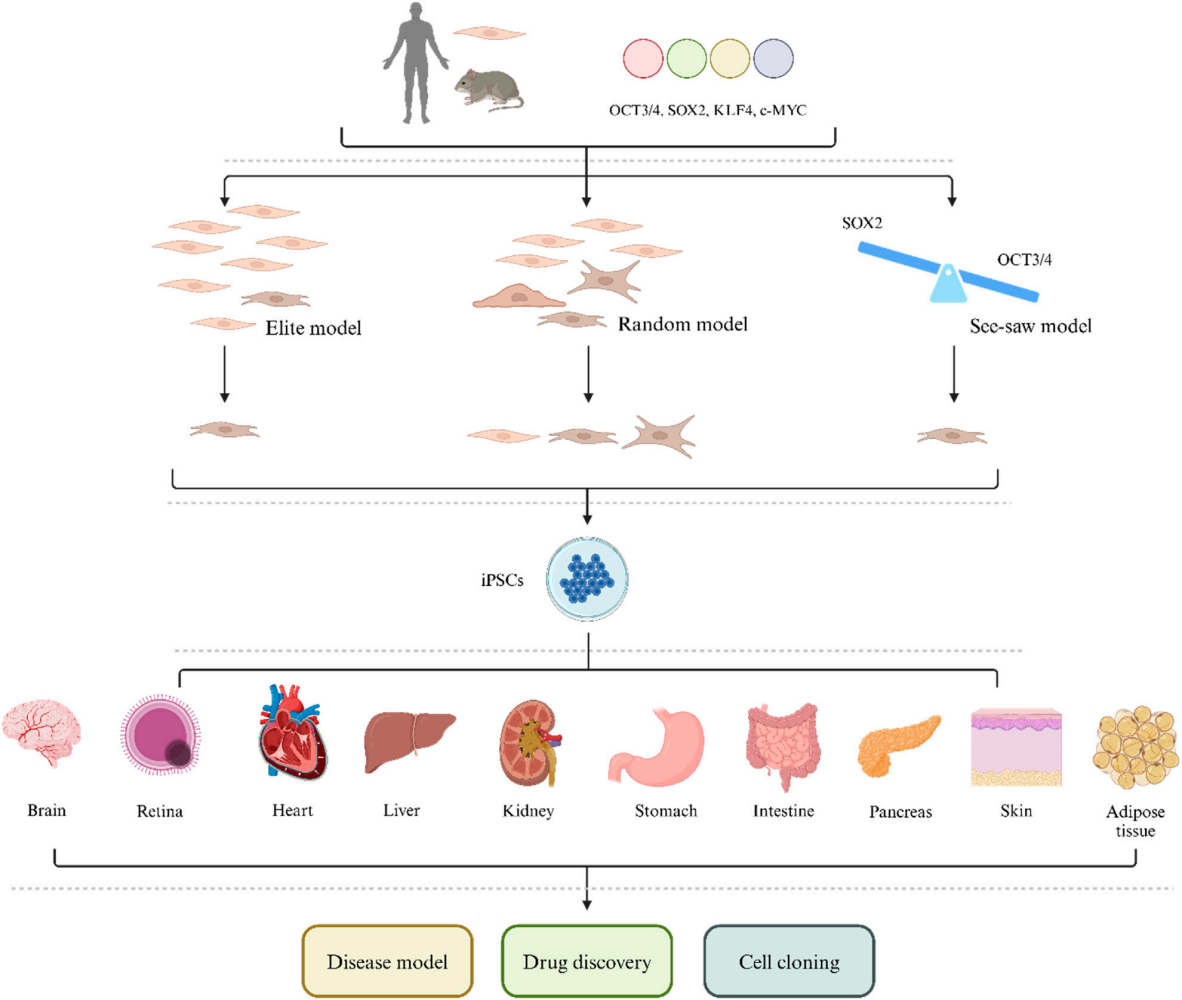
The mechanism and clinical application of iPSCs.

The first possible mechanism is the elite model (Figure 1) (Yamanaka, 2009). This model suggests that in a population, only a few elite cells, such as progenitor cells and stem cells (or cells with these characteristics), can be induced by pluripotent factors. In other words, only progenitor cells and stem cell populations can be transformed into iPSCs. However, lineage tracing studies and cloning analysis have shown that this model is not accurate and that iPSCs can be produced by ultimately differentiated cells, such as T and B lymphocytes, pancreatic β cells and albumin-expressing liver cells, which demonstrates that fully differentiated cells can also undergo reprogramming (Aoi et al., 2008; Hanna et al., 2008; Stadtfeld et al., 2008).

The second possible mechanism is a random model (Figure 1) (Yamanaka, 2009). This theory suggests that OSKM can reprogram all mediated somatic cells in a fixed or random manner. During the fixed reprogramming process, all cells follow exactly the same path and undergo reprogramming with a fixed latency. In random reprogramming, the transitions between states in the cell population are different, so cells undergo reprogramming with different incubation periods. Therefore, reprogramming takes place in several stages, and if a cell cannot complete any of these stages, the entire reprogramming process will collapse. The early stages of reprogramming involve the inhibition of somatic genes, mesenchymal transition to the epithelium, and metabolic changes from oxidative phosphorylation to glycolysis (Li et al., 2010; Samavarchi-Tehrani et al., 2010; Panopoulos et al., 2012; Zhang et al., 2012). The later stages include the activation of pluripotency-related genes, the inhibition of tissue-specific transcription factors and developmental genes, and a series of events, such as DNA and histone methylation (Banito et al., 2009; Hong et al., 2009; Kawamura et al., 2009). If any of these events are affected, the reprogramming path will be disrupted.

The third theory is the seesaw model, which emphasizes the importance of stoichiometry. In the early stages of reprogramming, OCT3/4 activates the expression of mesodermal genes and inhibits the expression of ectodermal genes (Papapetrou et al., 2009). However, SOX2 promotes gene expression in the ectoderm and reduces gene expression in the mesoderm (Yamaguchi et al., 2011). This step is important for further progress in reprogramming because it induces transient mesodermal features in intermediate products. To stably reprogram somatic cells into iPSCs, the levels of cell fate-determining factors need to be balanced. On the other hand, unfavorable OSKM expression causes intermediate cells to deviate from the reprogramming process (Polo et al., 2012; Tanabe et al., 2013). Some studies have reported that OSKM stoichiometry has a selective advantage in inducing the reprogramming of somatic cells into iPSCs (Yano et al., 1993; Carey et al., 2011; Shu et al., 2013). Wernig et al., 2008 reported that cells carrying a doxycycline-induced OSKM expression cassette had significantly greater reprogramming efficiency than did normal somatic cells. In addition, OCT3/4^high^SOX2^low^ stoichiometry is not only important for early ectopic expression but also has an indelible effect on late endogenous expression. In the later stages of reprogramming, when the transgenic gene is silenced in reprogrammed cells, activation of endogenous OCT3/4 is increased, while the expression of SOX2 remains at a low level (Buganim et al., 2012; Takahashi et al., 2014); therefore, improving reprogramming efficiency and regulating KLF4 expression can also achieve similar results (Kim et al., 2015). Surprisingly, transient expression of mesodermal genes was also observed in reprogrammed mouse and human cells in the later stages; this finding significantly advances research on reprogramming (Polo et al., 2012; Takahashi et al., 2014). Overall, the seesaw model indicates that the time and level of expression, as well as the stoichiometry of pluripotency factors, determine the pathway for reprogramming. According to this model, an imbalance in cell fate-determining factors will lead to unsatisfactory cell outcomes and the inability to reprogram somatic cells into iPSCs.

## Reprogramming methods

### Introduction of reprogramming factors through virus transduction

By integrating a retroviral vector into the cell surface, the OSKM gene is introduced into infected cells and integrated into the host genome, allowing cells to be reprogrammed and enter a pluripotent state (Polak et al., 2012; Jung et al., 2014; Ma et al., 2014; Gao et al., 2016; Kim Y. M. et al., 2017; Sayed et al., 2017; Verusingam et al., 2017; Moauro and Ralston, 2022). However, there is a significant risk of insertion mutations during the integration of retroviruses, which carry a significant carcinogenic risk. Although all primitive OSKM factors have some carcinogenic potential, studies have shown that the carcinogenic effect of c-Myc is particularly prominent (Nakagawa et al., 2008; Maekawa et al., 2011). Retroviruses can only be applied to dividing cells, which greatly limits their clinical application (Miller et al., 1990). The delivery of reprogramming factors through lentiviral vectors is another successful method that has greater reprogramming efficiency and less variability than the use of retroviruses (Jung et al., 2014; Gurusinghe et al., 2015; Chen et al., 2016; Abbey et al., 2019; Pessôa et al., 2019a; Gurusinghe et al., 2019; Khoshchehreh et al., 2019; Ruiz et al., 2019; Hernández-Sapiéns et al., 2020; Jiang et al., 2020; Chandrasekaran et al., 2021; Güney-Esken et al., 2021). Lentiviruses can transduce nondividing cells and exhibit selective tropism, which is conducive to high-level continuous expression of factors (Yu et al., 2007). However, there are still shortcomings, such as differences in preservation stability, small maximum insertion size, transgenic reactivation, and the safety of immunodeficient virus-derived lentiviruses (Patel and Yang, 2010; Schambach et al., 2013).

Adenovirus vectors reduce these risks, but they require high viral titers and repeated transduction, and reprogramming efficiency remains low (perhaps due to the dilution of reprogramming factors during cell growth and reproduction), making them difficult to apply in clinical practice (Kisby et al., 2021). Sendai virus (an RNA virus that does not integrate into the host genome) is a single-stranded RNA that replicates outside the cell nucleus and has been considered the safest viral method in recent years (Táncos et al., 2016a; Táncos et al., 2016b; Bueno et al., 2016; Chandrasekaran et al., 2016; Ochalek et al., 2016; Varga et al., 2016; Ma et al., 2017a; Terray et al., 2017a; Wang et al., 2017a; Ma et al., 2017b; Terray et al., 2017b; Ma et al., 2017c; Cristo et al., 2017; Ma et al., 2017d; Varga et al., 2017; Ahmed et al., 2018; Sanjurjo-Soriano et al., 2018a; Sanjurjo-Soriano et al., 2018b; Zhang et al., 2018; Erkilic et al., 2019a; Erkilic et al., 2019b; Pessôa et al., 2019b; Tarnawski et al., 2019; Sanjurjo-Soriano et al., 2022). This approach avoids the risks of insertion mutations, transgenic activation, and residual expression while having better reprogramming efficiency than the lentivirus method (Fusaki et al., 2009; Nishimura et al., 2011). However, although the Sendai virus is not pathogenic to humans, it may infect epithelial cells, so its application requires caution (Yonemitsu et al., 2000; Hu, 2014).

### Nonviral-mediated introduction of reprogramming factors

Compared to viral vectors, nonviral vectors allow cells to be reprogrammed without virus production and are not constrained by viral trends. This advantage makes this method safer and does not pose risks such as residual expression, genetically modified reactivation, insertion mutations of integrated viruses, or problems with the virus itself. First, the transduction of plasmids encoding reprogramming factors, including traditional plasmids, self-replicating exogenous plasmids, and microcyclic plasmids, can induce pluripotent stem cells (Okita et al., 2008; Fusaki et al., 2009; Hu, 2014). The Epsomal plasmid containing EBNA-1 and Orip sequences based on Epstein–Barr virus seems to have better application prospects (Yu et al., 2009; Hu and Slukvin, 2013; Hu, 2014). The plasmid was transfected into human cells to express the EBNA-1 protein, and the Orip sequence was subsequently recognized, inducing *in vitro* amplification of the plasmid. The plasmid has the ability to self-replicate and can achieve single transfection reprogramming (Yu et al., 2009). Unfortunately, traditional plasmids cannot replicate in mammalian cells and require multiple rounds of transfection for successful reprogramming, resulting in a much lower efficiency than that of viral vector methods. Compared to the plasmid method, the mini loop vector (a circular, supercoiled DNA element) has a longer expression time and stronger expression intensity in cells (Mayrhofer et al., 2009; Wasik et al., 2014). However, compared to the viral method, even if multiple consecutive transfections are performed, its reprogramming efficiency is still much lower.

Another nonviral method that introduces reprogramming factors is the PiggyBac transposon subsystem. A transposon is a DNA-based vector that catalyzes the removal and insertion of transposon enzymes within the genome. The PiggyBac transposon system was discovered in the cells of the beehive moth (Fraser et al., 1983). In mouse cell lines, when the reprogramming process no longer requires exogenous transgenic reprogramming factors, the transposons can be eliminated without tracing by secondary treatment with transposase, even if they have been integrated into the cell (Kaji et al., 2009; Woltjen et al., 2009). Because of this, the reprogramming steps are more complex, and the risk of incomplete excision and transposition insertion mutations is increased. In addition, the reactivation of transgenic genes is a problem that cannot be ignored. The human genome also contains components similar to the PiggyBac transposon subsystem, and it is currently unclear whether these components interact with the PiggyBac system (Sarkar et al., 2003; Hu, 2014).

The transfer of reprogramming factors through mRNA has also been explored (Durruthy Durruthy et al., 2014; Singh et al., 2015; Sayed et al., 2017; Ishtiaq et al., 2018; Bax et al., 2019; Giulitti et al., 2019; Lee et al., 2021). Warren et al., 2010 synthesized RNA encoding reprogramming factors using modified nucleotides and successfully induced iPSCs in human fibroblasts and peripheral blood. Compared with the other methods mentioned above, mRNA-based reprogramming is faster and more efficient and has a lower risk of mutation (Warren et al., 2010; Warren et al., 2012). However, exogenous mRNA can trigger a strong innate immune response, making it the main target of RNA-induced silencing complex (RISC) degradation. Therefore, the half-life of mRNA *in vivo* is very short, and the recombinant B18R protein of the vaccinia virus is used to minimize this negative impact (Warren et al., 2010). The disadvantages of this technology are the survival time of repeated transfections and the continuation of many modified, high-quality long sequence mRNAs. Significant efforts to optimize and improve efficiency are still needed.

Kim et al., 2009; Zhou et al., 2009 successfully delivered reprogrammed transcription factors in the form of proteins into mouse and human cells, although this process was inefficient and slow. Wasik and others have also successfully reprogrammed recombinant proteins produced in *Escherichia coli* (Hu, 2014; Wasik et al., 2014), but the proteins obtained from bacteria lack eukaryotic posttranslational modifications during regeneration, which may lead to misfolding and affect reprogramming efficiency. Additionally, the concentration of cell extracts derived from recombinant proteins produced in mammalian cells may still be low. The delivery of recombinant proteins can enable cell reprogramming without involving any exogenous nucleic acids or altering the genome, thus demonstrating good safety. If its efficiency can be further improved, then reprogramming transcription factors using proteins may become a good option.

In recent years, pluripotent reprogramming of mouse and human cells based on mature microRNAs (miRNAs) has become a promising approach (Chen et al., 2012; Wang G. et al., 2013; Yang and Rana, 2013; Ma et al., 2014; Fatima et al., 2016; Nguyen et al., 2017). MiRNAs are short noncoding RNAs, and the overexpression or knockdown of key genes with miRNAs during biogenesis can enhance or reduce the efficiency of reprogramming (Card et al., 2008; Choi et al., 2011; Leonardo et al., 2012; Guo et al., 2014; Wasik et al., 2014). The currently recognized explanation is that a single miRNA can regulate hundreds of targets, and introducing an miRNA cluster can naturally synergistically regulates thousands of targets (Lewis et al., 2005; Helwak et al., 2013; Guo et al., 2014). Direct transfection of mature miRNAs can avoid the negative effects of most other reprogramming methods; research by Anokye Danso revealed that the reprogramming efficiency is very high when viral vectors are used to introduce miRNA-encoded DNA (Anokye-Danso et al., 2011). However, the disadvantage of this method is that it requires multiple transfections, which are very expensive, and miRNAs may affect the activity of nonreprogrammed cells when regulating many targets.

According to current research, the efficiency of virus-mediated reprogramming is high, but it cannot overcome the adverse consequences of insertion mutations, transgenic reactivation, and residual expression, except for the Sendai virus. Other methods based on DNA, RNA, or protein can avoid these risks, but their efficiency does not meet expectations. For the various methods of reprogramming to be applied in clinical practice, their safety must be greater than their effectiveness. Initially, reprogramming was prohibited in clinical practice because the integration of the retroviral c-Myc genome increased the tumorigenicity of reprogramming (Nakagawa et al., 2008). With the advancement of technology and increasing research, Sendai viruses, episomal plasmids, DNA vectors, etc., have avoided the aforementioned risks, providing possibilities for the clinical application of reprogramming (Okita et al., 2011; Nishimura et al., 2017). At present, the negative impact of RNA or protein-based reprogramming is minimal, but the technology needs to be continuously improved to apply reprogramming to clinical practice (Durruthy and Sebastiano, 2015; Revilla et al., 2016). In addition, small-molecule compound-mediated reprogramming has the advantages of nontransgenic, nonexogenous nucleic acids and viral particles, and animal experiments have shown its efficiency to be acceptable (Hou et al., 2013). However, this method has not yet shown moderate efficacy in human cells, and small-molecule compounds can affect the related functions of epigenetic cell cycle regulation (Marión et al., 2009; Wiechec, 2011; Cieślar-Pobuda and Los, 2013). In the future, more research efforts are needed in this field.

## Factors affecting reprogramming efficiency

In response to the problem of low reprogramming efficiency, researchers have proposed many methods to improve reprogramming efficiency, including various enzymes, molecular compounds, and changes in the reprogramming environment.

### Protective effect of aging

Research has shown that aging seems to have an indelible relationship with reprogramming (Zhai et al., 2015; Ocampo et al., 2016; Mendelsohn et al., 2017; Mosteiro et al., 2018; Ghimire et al., 2020; Mas-Bargues et al., 2020; Alle et al., 2022; Singh and Zhakupova, 2022). The cycle induction of reprogramming genes is related to the induction and inhibition of aging genes. *In vivo* reprogramming requires the introduction of an inducible reprogramming cassette that allows the homogeneous expression of reprogramming factors. However, in physiological environments, tissue damage may accumulate through aging cells, creating a tissue environment conducive to neighboring cell reprogramming in the body, thereby improving reprogramming efficiency (Mosteiro et al., 2016). The result of reprogramming is “rejuvenation,” which goes against the aging process. Overall, there are two theories that suggest that OSKM (or other alternative molecules) can trigger the revival of organisms. One theory is that these factors reconnect the global chromatin landscape through embryonic means, thereby eliminating the epigenetic erosion caused by aging (Percharde et al., 2018; Della Valle et al., 2022). The second theory is that the expression of OSKM or chemicals promotes the encoding, storage, and recovery of epigenetic information from young adulthood in adult cells (Yang et al., 2023). Previous studies have shown that aging signals triggered by tissue damage and aging can promote the efficiency of reprogramming in the body (Mosteiro et al., 2016; Yang et al., 2023). This interaction may enhance the potential for partial reprogramming to maintain damaged and aged tissues. Mosteiro et al., 2016 reported that OSKM-induced aging requires the expression of the Ink4a/Arf locus and, through the production of the cytokine interleukin-6, creates a favorable tissue environment for *in vivo* reprogramming. Biological conditions related to aging, such as tissue damage or aging, are also beneficial for *in vivo* reprogramming of OSKM. Further research has shown that the Ink4a/Arf site and p53 pathway regulate *in vivo* reprogramming in an extracellular manner by generating environmental tissue aging and inflammatory responses (Mosteiro et al., 2018). The study by Ocampo et al. described the potential of short-term expression of OSKM to improve the recovery of aging tissue damage. Epigenetic remodeling during cell reprogramming improved age-related phenotypes, thereby demonstrating the important role of epigenetic dysregulation in driving aging in mammals (Ocampo et al., 2016). Yang et al., 2023 also reported that accurate DNA repair promotes aging at the physiological, cognitive, and molecular levels, including the erosion of epigenetic landscapes, extracellular differentiation, aging, and the DNA methylation clock, which can be reversed through OSKM-mediated regeneration. In transgenic OSKM-induced mice with p16INK4a/ARF deficiency, tissue senescence did not occur, which greatly inhibited reprogramming. The use of drugs that mimic the function of p16INK4a and increase cell aging led to increased levels of reprogramming, confirming the above results. Aarts et al. combined single-cell RNA sequencing (scRNA-seq) with short hairpin RNA (shRNA) screening to reveal a novel mechanism by which mechanistic target of rapamycin (mTOR) affects reprogramming and regulates aging. Inhibiting mTOR can inhibit the induction of cyclin-dependent kinase (CDK) inhibitors (CDKIs), including p16 (INK4a), p21 (CIP1), and p15 (INK4b), thereby preventing OSKM-induced aging (Aarts et al., 2017). Moreover, inhibiting mTOR weakens the senescence-associated secretory phenotype (SASP), which in itself is beneficial for reprogramming (Aarts et al., 2017). Downregulation of p53 can cause significant DNA damage within cells, leading to increased aging and increased production of cytokines such as IL-6, thereby improving reprogramming efficiency (Mosteiro et al., 2016). However, p53 is crucial for maintaining genomic integrity, especially since reprogramming itself can affect genomic integrity. It is currently unclear whether p53 knockout can be safely applied in clinical practice, and selecting specific mediators to target the p53 pathway may be a future research direction. In addition, Cheng et al., 2022 reported that reprogramming of degenerative intervertebral disc nucleus pulposus cells (NPCs) can reverse intervertebral disc degeneration (IDD) through short-term OSKM induction.

Although many studies have elucidated the relationship between aging and reprogramming, it is still unclear how changes in cellular aging signals promote the rejuvenation of the body, and whether aging cells can recover their vitality in a sustained manner is also unknown. The microenvironment, transgenic duration, and expression level may be key determinants of these processes, and the different properties of individual body weight programming stages and their relationships with aging may also have specific impacts. In the future, more research and new treatment strategies may be developed to improve diseases related to aging, provide new insights for regenerative medicine, achieve higher health standards for people, and even extend their lifespan.

### The impact of epigenetic barriers on reprogramming

**FIGURE 2:**
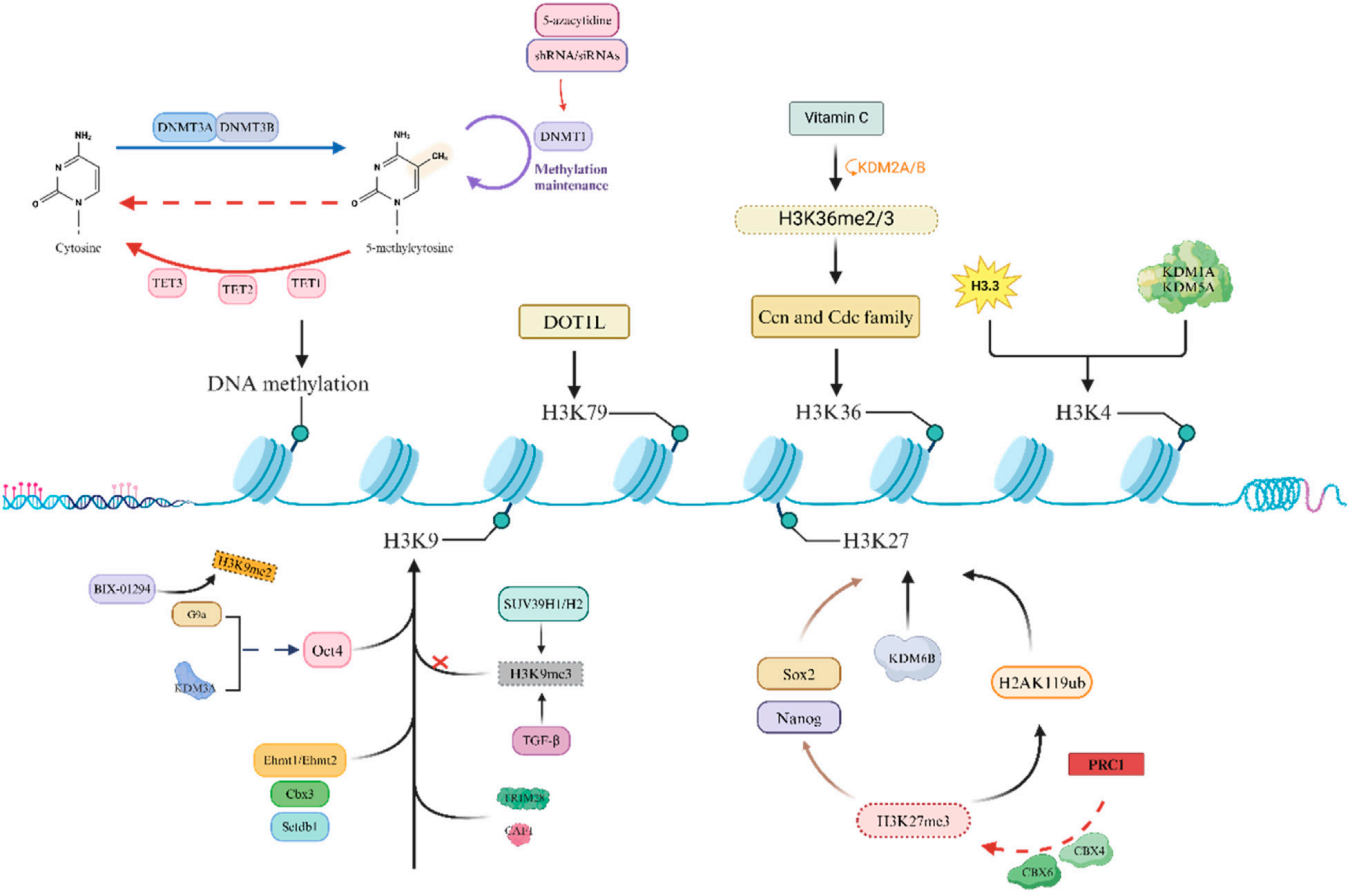
Epigenetic barriers on cell reprogramming.

Reprogramming is an epigenetic process that does not directly alter the DNA sequence. Genetic changes occur only when mutations develop during the reprogramming process or when transgenes are integrated into the genome. Pioneer factors such as POU5F1 (OCT4), NANOG, and SOX2 are transcription factors that can bind closed and preferentially methylated loci; therefore, reprogramming factors are powerful epigenetic remodelers of the somatic state by promoting the expression of normally inactive loci (Iwafuchi-Doi and Zaret, 2014). It has been confirmed that there is an important connection between epigenetic changes and reprogramming, and epigenetic-modifying factors play an indispensable role in reprogramming (Mao et al., 2017). However, the accumulation of epigenetic changes (such as acetylation or methylation) increases the risk of cancer, especially those associated with chronic inflammation (Bhattacharya et al., 2023; Chen et al., 2023; de Lima et al., 2023; Song et al., 2023). Some enzymes that regulate posttranslational modifications of histones can also promote cell fate toward pluripotency or differentiation by overexpressing or downregulating genes related to pluripotency, thereby affecting epigenetic modifications of transcription (Figure 2).

One of the main obstacles to OSKM-induced reprogramming is DNA methylation, and in some genomic regions where transcription is crucial, cells that cannot be demethylated in the later stages often cannot undergo reprogramming (Wang G. et al., 2017). Many studies have shown that DNA methyltransferase is an important factor in the reprogramming process (Chondronasiou et al., 2022b). In addition, Gao et al., 2018 reported that knocking down the newly formed methyltransferases Dnmt3a and Dnmt3b leads to the demethylation of genes related to totipotency, thereby improving reprogramming efficiency. Knocking down Dnmt3a and Dnmt3b can enhance the efficiency of OSKM-mediated reprogramming, while overexpressing both can inhibit reprogramming (Pawlak and Jaenisch, 2011; Guo et al., 2013). Mikkelsen et al., 2008 also reported that the instantaneous inhibition of DNA methyltransferase 1 (DNMT1) by small-molecule inhibitors such as 5-azacytidine or shRNAs/siRNAs promotes the expression of completely reprogrammed cells, and the downregulation of DNMT1 facilitates reprogramming to pluripotency. The second aspect is that the 10–11 translocation (TET) protein has been found to have an indelible role in DNA methylation. Research by Gao et al. suggested that TET1 promotes OCT4 demethylation and reactivation in early reprogramming and can even mediate OSKM reprogramming by replacing exogenous OCT4 (Gao et al., 2013). Chen et al. further demonstrated that the combination of TET1 and OCT4 can also achieve reprogramming, and the quality of mouse iPSCs produced is good (Chen et al., 2015). Furthermore, Sardina et al. reported that TET2 is recruited to the genomic regions required for iPSC reprogramming, where it promotes DNA demethylation before chromatin opening to mediate the precursor activity of Klf4 during reprogramming (Sardina et al., 2018). Gu et al., 2011 reported that SCNT embryos lacking TET3 exhibit increased levels of Oct4 promoter methylation. Overall, the regulation of DNA methylation plays an indispensable role in inducing pluripotency in cells. In summary, the dynamic regulation of DNA methylation plays a crucial role in inducing reprogramming.

The second major obstacle to reprogramming is the methylation of the heterochromatin marker H3K9, which limits the entry of reprogramming factors to some extent. Soufi et al. demonstrated that inhibiting SUV39H1/H2 (a methyltransferase responsible for H3K9 methylation) can improve reprogramming efficiency (Soufi et al., 2012). A study by Ma et al. suggested that knocking down histone methyltransferase G9a alone or overexpressing the H3K9 demethylase KDM3A can promote the reactivation of the Oct4 promoter (Ma et al., 2008). Epsztejn-Litman et al. also reported that G9a may inactivate many early embryonic genes by causing heterochromatin and *de novo* DNA methylation of H3K9 (Epsztejn-Litman et al., 2008). Further research revealed that the addition of BIX-01294 (a small-molecule inhibitor of G9a) can inhibit the expression of H3K9me2, thereby improving reprogramming efficiency (Epsztejn-Litman et al., 2008). Moreover, the knockout of Ehmt1, Ehmt2, Setdb1 (H3K9 transcript), and Cbx3 (a member of the heterochromatin protein 1 family) can also improve reprogramming efficiency (Sridharan et al., 2013). There are also studies showing that the inhibition of TGF-β after signal conduction decreases the signal intensity in the H3K9me3 region, thereby improving reprogramming efficiency. By adjusting the TGF-β activity at different reprogramming stages, the efficiency significantly improved (Wang et al., 2016). In addition, H3K9 methylation can recruit multiple proteins, such as tripartite motif containing protein 28 (TRIM28, a transcription inhibitor) and chromatin assembly factor complex (CAF1), which may become obstacles to reprogramming (Sripathy et al., 2006; Cheloufi et al., 2015). Therefore, the dynamic regulation of H3K methylation plays a crucial role in improving reprogramming efficiency.

In addition, research has shown that some histones also play irreplaceable roles in the context of development and reprogramming. Onder et al., 2012 showed that the CBX protein within the polycomb repressor complex (PRC1) can recognize H3K27me3, which in turn catalyzes the monoubiquitination of histone H2A lysine 119 (H2AK119ub), leading to transcriptional inhibition. Moreover, H3K27 methyl readers within PRC1 (such as CBX4 and CBX6) reduce reprogramming efficiency by inhibiting the pluripotency genes Sox2 and Nanog (Ning et al., 2017). Inhibiting the expression of EZH1 in mouse reprogramming can enhance the efficiency of inducing pluripotency (Cacchiarelli et al., 2015); in contrast, overexpression of EZH2 helps to induce pluripotency through the mesenchymal to epithelial transition (MET) (Buganim et al., 2012; Ding et al., 2014; Rao et al., 2015), which may be related to the fact that the two subunits have different targets. Paradoxically, some studies have shown that when the H3K27 demethylase KDM6a/KDM6b in mouse embryonic fibroblasts (MEFs) is knocked down, reprogramming efficiency is significantly improved (Mansour et al., 2012; Zhao et al., 2013).

An increasing number of studies have shown that factors related to active transcription can also hinder reprogramming by maintaining somatic expression programs. Inhibiting these barriers has been shown to effectively improve reprogramming efficiency. DOT1L mediates H3K79 methylation, and its genetic and pharmacological inhibitory effects can improve reprogramming efficiency and promote the production of pluripotent stem cells (CiPSCs) induced by mouse somatic cell chemistry. Previous studies have shown that DOT1L appears to play a role only in the early stages of reprogramming (Khoshchehreh et al., 2019), and inhibition of DOT1L activity has been shown to play a very significant role in improving reprogramming efficiency (Ichida et al., 2014; Jackson et al., 2016; Tran et al., 2019). Other studies have shown that H3K79 demethylation can significantly enhance reprogramming by appropriately stimulating FOXH1 expression (Takahashi et al., 2014; Wang et al., 2019a). In addition, Wang et al. demonstrated that ascorbic acid (vitamin C, a cofactor of histone demethylase) can induce H3K36me2/3 demethylation through KDM2A/B (an H3K36 demethylase), leading to the upregulation of key cell cycle regulatory factors such as Ccn and Cdc family genes, thereby improving the efficiency of inducing pluripotency (Wang et al., 2011). Moreover, the addition of ascorbic acid can inhibit the Ink4/Arf site, which may lead to cells bypassing OSKM-induced aging, thereby enhancing the acquisition of pluripotency (Wang et al., 2011). However, not all histone methylation inhibits reprogramming. Cacchiarelli and Dabiri et al. reported that inhibiting the expression of the H3K4 demethylases KDM1A and KDM5A can promote the production of iPSCs (Cacchiarelli et al., 2015; Dabiri et al., 2019). Interestingly, a mutant histone H3.3 has also been shown to be an obstacle to the induction of reprogramming by blocking the acquisition of H3K4 methylation (Jullien et al., 2017; Mor et al., 2018).

In addition to the factors listed above that hinder reprogramming, proteins related to transcription mechanisms can also serve as obstacles to reprogramming. One example is RNA polymerase II-associated protein 1 (RPAP1), which can promote gene transcription related to cell identity through the interaction of RNA polymerase II (RNA Pol II)/mediator (Whyte et al., 2013; Allen and Taatjes, 2015). Lynch et al., 2018 also reported that the loss of RPAP1 in shRNA-mediated MEFs leads to the loss of mesenchymal cells and fibroblasts and promotes early reprogramming induced by OSKM.

### Cell survival environment

The cultivation conditions for iPSCs also play an indispensable role in reprogramming. For the cultivation of human pluripotent stem cells, both feeder-free (Ff) and xeno-free (Xf) culture conditions are necessary (Nakagawa et al., 2014). Xu et al., 2001 breakthrough study used Matrigel (a heterologous substrate) as a substitute for MEF feeding, using laminin as a binder, to demonstrate an Ff system for amplifying human PSCs. Subsequently, Ludwig et al. described TeSR1, a five-cocktail culture medium, as the first Ff-Xf system and subsequently discovered an eight-cocktail culture medium for cultivating PSCs using a vibrational linker protein under Ff-Xf conditions (Ludwig et al., 2006; Chen et al., 2011). Miyazaki et al., 2012 recently discovered a shorter laminin-511 (a cell adhesion molecule compatible with the Ff system that promotes the growth of human iPSCs) active fragment, laminin-511 E8 (LN511E8), that can increase adhesion and effectively maintain human ESCs and iPSCs. Human iPSCs can be isolated into individual cells and plated on a culture plate coated with the recombinant LN511E8 protein (rLN511E8). Compared to other matrices, it can more effectively form colonies. Moreover, rLN511E8 is easier to extract, more pure, and less expensive than the full-length laminin-511 protein (Yamada and Sekiguchi, 2015). At present, the combination of rLN511E8 and StemFit (an Xf medium) works well as an Ff-Xf system. It can stably label gene expression and induce the production of iPSCs (Nakagawa et al., 2014). In addition, many other materials, including recombinant proteins and synthetic polymers, can replace feeder cells (Mei et al., 2010; Rodin et al., 2010; Lu et al., 2012).

In addition to the above culture conditions, the composition of the microenvironment around cells can affect the efficiency of somatic reprogramming (Liu et al., 2021). Two studies have shown that cultivation under hypoxic conditions can promote the generation of iPSCs, which has been confirmed in both human and mouse cell experiments (Yoshida et al., 2009; Cieślar-Pobuda et al., 2015). Forristal et al. also confirmed that hypoxia inducible factors (HIFs) can regulate the expression of the three most commonly used reprogramming factors, Oct4, Sox2, and NANOG, in cultured human embryonic stem cells under hypoxic conditions (Forristal et al., 2010).

### Factors that can promote reprogramming

Moreover, some unique compounds and cytokines can also affect the efficiency of reprogramming. Seo et al., 2022 reported that a flavonoid compound, licorice chalcone D (LCD), which is mainly present in liquorice roots, can enhance the generation of iPSCs in somatic cells by promoting MET in the early stages of reprogramming. The results of Kim K. M. et al., 2017 indicated that grass root soup (SGT-4) significantly improved the efficiency of human iPSC generation through OSKM. Lee et al., 2020 demonstrated that the activation of mTOR significantly enhances the production of iPSCs in human somatic cells with ectopic OSKM expression. Overactivated endoplasmic reticulum (ER) stress can hinder the initial steps of MET, thereby hindering the formation of iPSCs in mesenchymal cells (Fuentes-Iglesias et al., 2022). The study by Feng et al., 2022 showed that in the early stages of reprogramming and iPSC generation, knocking down Sin3a significantly disrupts MET, and disrupting the interaction between Sin3a and Tet1 can significantly block the generation of MET and iPSCs. Wu et al., 2021 demonstrated that when coexpressed with OSKM, Surf4 can activate the response to ER stress in the early stages of reprogramming, significantly promoting the generation of iPSCs without relying on proliferation. Wang et al., 2021 showed that short-term induction of the local expression of OSKM in muscle fibers can promote the activation of muscle stem cells or satellite cells (SCs), thereby accelerating muscle regeneration in young mice, which may promote tissue regeneration by altering the stem cell niche. Wu et al., 2017. Demonstrated that the oocyte-specific factor Obox1 strongly activated somatic cell reprogramming by promoting MET and reducing excessive cell proliferation. In addition, CM272 can promote the generation of human iPSCs by removing the strongest carcinogenic factor, c-Myc (Rodriguez-Madoz et al., 2017). Wang et al., 2019b reported that the synergistic effect of NANOG and LIN28 (NL) can increase OSKM-mediated reprogramming by approximately 76-fold and shorten the reprogramming delay by at least 1 week. This synergistic effect is inhibited by GLIS1 but enhanced by histone methyltransferase DOT1L inhibitors (iDOT1L). Further research revealed that LIN41 can replace LIN28 and synergize with NANOG, and under WNT inhibition, the coexpression of LIN41 and NL further promotes the formation of mature iPSCs (Wang et al., 2019b). Zhou et al., 2016 reported that as reprogramming begins, reactive oxygen species (ROS) generation significantly increases. The consumption of ROS through antioxidants or Nox inhibitors significantly reduces reprogramming efficiency, while knocking down and knocking out p22 (phox) (a key subunit of nitrogen oxide (1–4) complexes) reduces reprogramming efficiency. However, excessive ROS generated using genetic and pharmacological methods also impair reprogramming. This suggests that an optimal level of ROS signaling is crucial for inducing pluripotency. Di Stefano et al. reported that in primary B cells of mice, transient C/EBPα expression and OSKM activation induce a 100-fold increase in the reprogramming efficiency of iPSCs, involving 95% of the population (Di Stefano et al., 2014). During this transformation process, pluripotency and epithelial mesenchymal transition genes were significantly upregulated, and 60% of the cells expressed Oct4 within 2 days. C/EBPα also induces the expression of the dioxygenase Tet2 and promotes its translocation to the nucleus, where it binds to the regulatory region of pluripotent genes and becomes demethylated after OSKM induction (Di Stefano et al., 2014). Moreover, overexpression of Tet2 enhances OSKM-induced B-cell reprogramming (Di Stefano et al., 2014). Rais et al., 2013 demonstrated that Mbd3, a core member of the Mbd3/nucleosome remodeling and deacetylation (NuRD) repressor complex, coupled with OSKM transduction and reprogramming under initial pluripotency promotion conditions leads to deterministic and synchronous iPSC reprogramming (nearly 100% efficiency within 7 days in mouse and human cells). Pijnappel et al. reported that knocking down the transcription factor IID (TFIID) complex affects the pluripotency circuit of mouse embryonic stem cells and inhibits the reprogramming of fibroblasts. The TFIID subunit forms a feedforward loop with the OSKM factors, inducing and maintaining a stable transcription state, and the transient expression of the TFIID subunit greatly enhances reprogramming (Pijnappel et al., 2013). Huynh et al. showed that the histone variants TH2A and TH2B and the histone chaperone nuclear fibrinolytic protein (NPM2), which are enriched in oocytes, enhance OSKM-induced reprogramming of adult and neonatal human dermal fibroblasts and umbilical vein endothelial cells and improve the quality of human iPSCs (Huynh et al., 2016). Ke et al., 2017 emphasized the crucial role of CX45 in reprogramming and its potential to increase the cell division rate and accelerate the kinetics of iPSC generation. Wang et al., 2020 concluded that TFAP2C serves as a strong activator of somatic reprogramming by promoting MET and inhibiting c-Myc-dependent apoptosis. Oh et al., 2016 demonstrated that cyclin D1 is an essential gene in the reprogramming process, and its activation by reprogramming factors is an important process in somatic reprogramming. Chen et al., 2016 reported that the use of the histone deacetylase inhibitor sodium valproate (VPA) during reprogramming can improve the induction of iPSCs. Zhao et al. also emphasized the role of VPA in breaking the cellular aging barrier to induce pluripotency (Zhai et al., 2015). Wei et al. reported a new chemical, CYT296, that can increase the ability of OSKM-mediated induction of iPSCs 10-fold, and efficient reprogramming can be achieved by combining Oct4 with other small molecules (Wei et al., 2014). They also proposed a new method to regulate somatic reprogramming by targeting small molecules involved in chromatin deconcentration. Using OSKM, Declercq et al. showed that Zic3 not only improved reprogramming efficiency but also significantly reduced the number of clones generated during iPSC generation (Declercq et al., 2013). In addition, Melendez et al. reported that natural killer (NK) cells significantly limit reprogramming both *in vitro* and *in vivo* (Melendez et al., 2022). On the other hand, Recchia et al.'s study demonstrated that cell line origin and cell proliferation rate are also determining factors for cell reprogramming into pluripotency (Recchia et al., 2022). Furthermore, Xu et al. unexpectedly observed that removing c-Myc from the combination of OSKM greatly enhanced the generation of iPSCs. IPSCs without c-Myc exhibit significant pluripotency and can generate full-term mice through tetraploid complementation (Xu et al., 2013). Interestingly, Kim et al. reported for the first time that mechanical stimulation can improve reprogramming efficiency without increasing infection rates (Kim Y. M. et al., 2017).

## Cancer risk

Instantaneous reprogramming can promote epigenetic changes and eliminate the expression of various markers of the aging phenotype, but it is not sufficient to induce endogenous pluripotency markers or loss of cellular identity. Therefore, theoretically speaking, partial reprogramming can delay or even eliminate the accumulation of aging phenotypes without causing cancer. Unfortunately, two of the OSKM reprogramming factors, c-Myc and Klf4, are oncogenes themselves, and their expression typically increases in metastatic cancer; Oct4 and Sox2 are also closely related to cancer (de Lázaro et al., 2017; Wang et al., 2019c; Ruiz et al., 2019).

Oct4 plays a crucial role in the reprogramming process; however, its promotion of pluripotency also induces the development of cancer. Research has shown that the overexpression of Oct4 alone can lead to poor development in mice (Hochedlinger et al., 2005). In addition, in breast cancer, the expression of Oct4 in cancer tissue is significantly increased, and Oct4 is considered a key factor in cancer occurrence and growth (Wang and Herlyn, 2015). Kim et al. reported that Oct4 is expressed in biochemically disrupted cancer stem cells (BCSCs) but not in non-BCSCs (Kim S. Y. et al., 2013; Bliss et al., 2018). Another study confirmed that HIF2α can directly bind to the Oct4 promoter to increase Oct4 transcription, thereby increasing the proportion of ALDEFLUOR-positive BCSCs (Kim R. J. et al., 2013). Asadi et al., 2011 also reported that Oct4 is associated with decreased differentiation and increased tissue invasion in gastric cancer, which can lead to a poorer prognosis. Various examples indicate that Oct4 induces reprogramming while also increasing the risk of cancer.

Sox2 can form heterodimers with Oct4 to activate genes involved in maintaining pluripotency (Lefebvre et al., 2007). This heterodimer is also overexpressed in multiple cancers, such as liver cancer, squamous cell carcinoma, and neuroblastoma (Islam et al., 2015; Fatma and Siddique, 2021). Santini et al. reported that Sox2 is a key factor in the self-renewal and tumorigenicity of melanoma cells (Santini et al., 2014). Wang et al. also demonstrated that OSKM can promote cell proliferation in melanoma cells by upregulating JAK2 and Cyclin-B1 (Wang et al., 2019c). Piva et al. reported that the level of Sox2 was greater in patients with endocrine resistance in breast cancer, and the high expression of Sox2 in breast cancer was associated with a low survival rate (Piva et al., 2014). Further research has shown that Sox2 silencing can also affect the formation of breast cells (Mukherjee et al., 2017).

Interestingly, in terms of carcinogenesis, Klf4 may have dual functions as a tumor suppressor and oncogene, depending on the type of cancer. It is highly expressed in more than 70% of breast cancer patients and is necessary to maintain breast cancer stem cells (Yu et al., 2011). Cittelly et al. showed that Klf4 was overexpressed in CD44-positive MCF-7 and T47D breast cancer cells, and downregulation of siRNA or miR-29 targeting Klf4 led to a decrease in the number of these cells (Cittelly et al., 2013). Similarly, Okuda et al., 2013 reported that overexpression of Klf4 in MDA-MB-231 cells increased the proportion of CD44^+^/CD24^-/low^/EpCAM^+^- CSC populations, while miR-7 targeting of Klf4 resulted in a significant reduction in this population. In addition, Klf4 was found to be involved in the brain metastasis of MDA-MB-231 cells (Liu et al., 2016). On the other hand, Leng et al. reported that Klf4 is overexpressed in colon cancer stem cell populations, and a decrease in its expression reduces the ability of these cells to produce tumors (Leng et al., 2013). At present, there is still little research on the tumor inhibitory effect of Klf4, and more results are needed to support this finding.

C-Myc is a recognized oncogenic gene that can increase tumor formation, and its expression is elevated in various cancers (Xiao et al., 2016). Research has shown that it plays an indispensable role in tumors that are prone to reprogramming (Senís et al., 2021). c-MYC endows hepatocellular carcinoma (HCC) cells with a malignant phenotype (Nio et al., 2017). Further research by Cheng revealed that the highly conserved oncogenic long chain noncoding RNA (THOR) lncRNA associated with testes β-catenin regulates c-MYC and participates in the dedifferentiation of HCC cells into HCC stem cells (Cheng et al., 2019). A study by Okita et al., 2007 suggested that 20% of tumors formed by iPSC-derived cells can be attributed to reactivation of c-Myc transgenic cells. Luan et al., 2022 demonstrated that upregulation of the MUC1/c-MYC pathway leads to poor prognosis in pancreatic ductal adenocarcinoma (PDAC). Lee et al., 2017 also reported that c-Myc can regulate the expression of BMI-1 (b-lymphoma Moloney murine leukemia virus insertion region-1) in breast cancer cells through transcription. Nakagawa et al., 2008 demonstrated that adult dermal fibroblasts can still undergo reprogramming without ectopic expression of c-Myc, indicating that c-Myc is not necessary for reprogramming. However, the absence of c-Myc greatly limits the efficiency of reprogramming.

In addition to the four key transcription factors mentioned above, the use of retroviruses and lentiviral vectors in some reprogramming methods also carries the risk of insertion mutations, which cannot be avoided. Compared with ESCs, iPSCs form teratomas faster, more efficiently, and more easily *in vivo* (Gutierrez-Aranda et al., 2010). In addition, iPSCs proliferate in an uncontrolled manner, similar to cancer cells, so transplanting iPSCs containing any residual iPS carries a risk of tumor formation (Gore et al., 2011). In addition, reprogramming may also trigger intracellular stress response pathways, which increase susceptibility to gene mutations. The production of iPSCs also requires multiple cell divisions, so gene mutations may accumulate during this process (Hussein et al., 2011; Laurent et al., 2011).

We discussed in the previous section that using nonviral vector-mediated reprogramming can effectively avoid the risk of insertion mutations, but its efficiency in inducing reprogramming is unsatisfactory. Notably, Li et al., 2011 reported that the addition of the glycogen synthase kinase 3 (GSK3) inhibitor CHIR 99021 can achieve reprogramming solely through Oct4 and Klf4, which may reduce the risk of tumorigenesis. Unfortunately, the experiment has been successful only in MEFs, and the desired results have not been achieved in human cells. Furthermore, even if reprogramming with only Oct4 and Klf4 can be successful in human cells, the remaining two factors related to cancer development still exhibit overexpression. Therefore, effectively reducing cancer risk still requires ongoing efforts. Cota et al. proposed a new viewpoint that in terms of speedier reprogramming of the required cells, transdifferentiation of a completely differentiated cell state directly into another differentiated cell state avoids the drawbacks of fully reprogramming cells to iPSCs (Cota et al., 2020). By bypassing the iPSC stage, transdifferentiation also decreases the chance of tumor formation (Cota et al., 2020).

## Not all OSKM factors are equally necessary

As the understanding of and research on reprogramming increases, OSKM can be used to successfully induce iPSCs, but an increasing number of substitutes have been discovered and prepared (Di Stefano and Graf, 2016; Xiao et al., 2016). Shu et al. reported that in the absence of OCT4 and SOX2, chemical screening can guide corresponding lineage specifications and induce pluripotency (Shu et al., 2013). Two years later, they showed that the GATA family was the first protein family in which all members could act as inducers of reprogramming processes, replacing Oct4 (Shu et al., 2015). Guan et al. demonstrated the chemical reprogramming of human somatic cells into CiPSCs by creating an intermediate plasticity state (Guan et al., 2022). This is the first case of chemical reprogramming in which small molecules from human cells replaced all OSKM reprogramming factors. A recent study revealed that, compared with OSKM, GATA3, OCT4, KLF4, and MYC (GOKM) can effectively generate induced trophoblast stem cells (iTSCs) from fibroblasts with pluripotent gene knockout, which seems to reprogram the chromatin of human fibroblasts better than OSKM does, further emphasizing that pluripotency is essential for obtaining iTSCs (Naama et al., 2023). Moreover, knocking down Wdr82 can significantly reduce the efficiency of somatic reprogramming. Further research has revealed that the molecular mechanism underlying this effect involves inhibition of mitochondrial oxidative phosphorylation (Cui et al., 2023). Ye et al. reported that the transcription factor LIM and cysteine-rich domain 1 (LMCD1), together with OSKM, can more effectively induce the reprogramming of human skin fibroblasts into iPSCs than can OSKM. Mai et al., 2018 revealed that NKX3-1 (a prostate-specific tumor suppressor) can replace exogenous OCT4, reprogramming mouse and human fibroblasts with considerable efficiency and producing fully pluripotent stem cells. Fritz et al., 2015 reported that in the absence of OCT4, several pathways (such as the Notch, Smoothened, and cAMP pathways) can generate alkaline phosphatase-positive colonies, and the activation of cAMP signaling can functionally replace OCT4 to induce pluripotency. Deng et al., 2015 used microRNA 302–367 to replace oncogenic Klf4 and c-Myc in OSKM as a safer strategy to successfully induce the generation of pluripotent stem cells. CPEPS-OS-miR, a type of nanoparticle, was used to prepare iPSCs from human umbilical cord mesenchymal stem cells with an efficiency more than 50 times greater than that of any single or possible combination of these factors (Oct4, Sox2, or miR-302–367). Buganim et al., 2014 reported that ectopic expression of Sall4, Nanog, Esrrb, and Lin28 (SNEL) in MEFs was more effective at producing high-quality iPSCs than other factor combinations, including OSKM.

## Clinical application of reprogramming

It has been more than 10 years since the discovery of the first generation of mouse iPSCs. In recent years, with the progress of research and technological advancements, the quality of iPSCs produced and the efficiency of reprogramming have also been greatly improved. The methods of reprogramming have gradually matured, and this technology has also begun to slowly demonstrate potential for clinical application.

### Disease model

To date, iPSCs have been used to study various neurological diseases (Kwak et al., 2020; Han et al., 2021), including amyotrophic lateral sclerosis (ALS) (Dimos et al., 2008; Kabashi et al., 2010; Egawa et al., 2012), Alzheimer’s disease (AD) (Táncos et al., 2016a; Chandrasekaran et al., 2016; Ochalek et al., 2016; Hernández-Sapiéns et al., 2020; Raska et al., 2021a; Raska et al., 2021b), and Parkinson’s disease (PD) (Ma et al., 2017a; Wang et al., 2017a; Ma et al., 2017b; Ma et al., 2017c; Ma et al., 2017d). Dimos et al. obtained the first human iPSCs from middle-aged and elderly ALS patients (Dimos et al., 2008), and Egawa et al. demonstrated that motor neurons generated from iPSCs from patients with TDP-43 mutations can form cytoplasmic aggregates typical of postdeath ALS neurons (Egawa et al., 2012). Chen et al., 2014 also reported that in ALS patients with superoxide dismutase 1 (SOD1) gene mutations, only motor neurons differentiated from iPSCs exhibited cytoplasmic aggregation. In an iPSC model of AD patients, neurons were successfully generated from two familial AD patients with APP gene duplication, two sporadic AD patients, and two healthy controls (Israel et al., 2012). Nelson et al. found that the APOE-R136S mutation prevented APOE4-driven AD pathology, neurodegeneration, and neuroinflammation using a human iPSC-derived neuron model (Nelson et al., 2023). Jiang et al. reported that dopaminergic neurons generated from the iPSCs of PD patients with Parkin mutations exhibited increased oxidative stress and dopamine efflux (Jiang H. et al., 2012). Mutation in GBA1, the gene encoding glucose cerebrosidase (GCase), is the most common genetic risk factor for PD, as demonstrated by Baden et al. using neurons derived from iPSCs. β-GCase can recognize internal mitochondrial-targeted sequences, such as signals from the cytosol to mitochondria (Baden et al., 2023). However, most current iPSC models are only isolated neurons, so the reasons for cellular nonautonomy are still undetermined. The main risk factor for many neurodegenerative diseases is age, which requires more time to develop in cell models, increasing the labor and cost of iPSC research. In the future, research needs to focus on these aspects.

Although there are many applications of animal models of cardiovascular disease (Kisby et al., 2021; Pushp et al., 2021), there are also multiple differences in ion channel characteristics and electrophysiology between human and mouse hearts (Davis et al., 2011). Therefore, this approach provides a new option for using iPSCs to study human cardiovascular diseases. A previous study reported a unique reprogramming strategy that involves regulating resident adult myocardial cell identity to an immature proliferative state (Chen et al., 2021). Sun et al., 2012 used iPSCs to simulate dilated cardiomyopathy (DCM) and reported that the addition of metoprolol improved sarcomere disorder caused by cells from patients with the R173W mutation in the TNNT2 gene. These abnormalities in actin structure were exacerbated by adrenaline receptor stimulation and improved after the addition of receptor blockers. Lan et al., 2013 studied iPSC-derived cardiomyocytes from hypertrophic cardiomyopathy (HCM) patients with MYH7 gene (R633H or R442G) mutations (Han et al., 2014). The mutated cardiomyocytes exhibited a greater frequency of sarcomere disorder and increased cell size, while treatment with the histone deacetylase activity inhibitor trachomycin A improved the disease phenotype. Another iPSC-HCM study used high-speed video imaging to visualize endothelin, a vasoconstrictor, which enhances the pathological phenotype (Tanaka et al., 2014). There have been many reports of heart rate disorders, including the iPSC model of long QT syndrome (Moretti et al., 2010; Lahti et al., 2012; Liang et al., 2013; Ma et al., 2013; Terrenoire et al., 2013; Matsa et al., 2014). Cristo et al. successfully induced the production of human iPSC lines from exfoliated renal epithelial (ERE) cells in patients with congenital heart disease (CHD) and unilateral defects (Cristo et al., 2017). The established iPSC line exhibits specific heterozygous changes, a stable karyotype, and the expression of pluripotent markers and produces embryoid bodies that can differentiate into three germ layers *in vitro* (Cristo et al., 2017).

The current understanding of cellular pathophysiology in hematology largely depends on the primary hematopoietic cells derived from patients and animal models. However, species differences limit the use of animal models, and the quantity obtained is also limited. Therefore, a blood disease model based on iPSCs is highly important. Raya et al., 2009 reprogrammed fibroblasts from patients with Fanconi anemia (FA), an autosomal recessive pediatric disease, and successfully established the first blood disease model based on iPSCs. Wang et al., 2009 reported that after gene correction, iPSCs obtained from patients with thalassemia differentiated into hematopoietic progenitor cells (Wang et al., 2012). Transplanting these progenitor cells into a mouse model restored human hemoglobin levels, which is a valuable combination of iPSC technology and homologous recombination gene correction. Gandre-Babbe et al., 2013 prepared iPSCs from malignant cells of two patients with juvenile myelomonocytic leukemia (JMML) with PTPN11 mutations and used these cells for drug screening, identifying MEK kinase inhibitors that may have therapeutic effects.

iPSCs have also been used to examine affected tissues from patients with some congenital immunodeficiency diseases. Lafaille et al., 2012 generated iPSCs from TLR3-or UNC93B-deficient patients and differentiated them into neuronal lineages to analyze the cellular autonomous immune responses in the central nervous system. Ciancanelli et al., 2015 reported that the number of type I interferons produced by lung epithelioid cells from patients with iPSCs decreased, and the replication of influenza virus increased. A study by Morishima et al., 2014 suggested that genetic correction of HAX1 in iPSCs in patients with severe congenital neutropenia can improve defective granulocyte production. Güney-Esken et al. successfully generated different iPSC clones from patients with Gricelli syndrome type 2 (GS-2), a rare autosomal recessive immunodeficiency syndrome caused by a mutation in the RAB27A gene (Güney-Esken et al., 2021). Jiang et al. also successfully established a model of chronic granulomatosis using iPSCs to screen candidate drugs and develop gene therapy (Jiang Y. et al., 2012). These studies demonstrate that IPSC-based modeling has a very effective role in examining the inherent defects of immune responses in specific organs or tissues.

With the increasing abundance of research, the discovery of human iPSCs has led to the creation of cells that can serve as *in vitro* models for many diseases (Táncos et al., 2016b; Bueno et al., 2016; Fatima et al., 2016; Varga et al., 2016; Terray et al., 2017a; Wang et al., 2017a; Terray et al., 2017b; Wang et al., 2017c; Varga et al., 2017; Ahmed et al., 2018; Sanjurjo-Soriano et al., 2018a; Sanjurjo-Soriano et al., 2018b; Kavyasudha et al., 2018; Zhang et al., 2018; Bax et al., 2019; Giulitti et al., 2019; Nagel et al., 2019; Castel et al., 2020). However, the established somatic cell populations have individual differences in maturity and function, which may be attributed to factors such as the origin of iPSCs, the presence of residual transgenes in each iPSC clone, interclone genetic variation, X chromosome inactivation status, and epigenetic modifications. These issues pose obstacles to accurately assessing disease phenotypes. These difficulties must be addressed to generate more accurate disease models based on iPSCs.

Interestingly, Guo et al. proposed an interruption reprogramming strategy to generate induced progenitor-like (iPL) cells from alveolar type II epithelial (AEC-II) cells. Interrupting reprogramming can lead to the controlled expansion of cell numbers but preserves the pathway for differentiation into alveolar epithelial cell lines (Guo et al., 2018). After the transplantation of AEC-II-iPL cells into injured lungs, the cells remain in the lungs and improve bleomycin-induced pulmonary fibrosis (Guo et al., 2018). Interrupted reprogramming can serve as an alternative method to generate highly specific functional therapeutic cell populations, which may lead to significant advances in regenerative medicine.

### Treatment and drug discovery based on iPSCs

Using disease models established by iPSCs as mentioned above, early disturbances that mark the development of the disease, which cannot be detected using other patient specimens, can be identified. These models can also be used for early intervention and drug screening, which will assist in the discovery of more new drugs and therapies for treating multiple refractory diseases (Al Abbar et al., 2020). Some clinical trials of ESC-based treatments are ongoing, such as studies of patients with diabetes, PD, and myocardial infarction, but with the development of iPSC technology, the therapeutic potential of this treatment is expected to greatly expand (Pushp et al., 2021). We have summarized the current clinical trials involving iPSCs in Table 1.

**TABLE 1:** Current clinical trials involving iPSCs.

Disease/Disorder	Drug	iPSC-derived cell type	Reference nos
**Amyotrophic lateral sclerosis**	Ropinirole	Motor neurons	Morimoto et al. (2023)
**Lung cancer/Head and neck cancer**	Autologous NKT cell	Natural killer T cell	Aoki and Motohashi (2023)
**Huntington’s disease**	Branaplam	Cortical neurons	Krach et al. (2022)
**Alloimmune platelet transfusion refractorines**	iPLAT1	Megakaryocyte	Sugimoto et al. (2022)
**Alzheimer’s disease**	Bromocriptine	Neuronal cell	Kondo et al. (2021)
**Steroid-resistant acute graft *versus* host disease**	CYP-001	Mesenchymal stromal cell	Bloor et al. (2020)
**Pendred syndrome**	Sirolimus	Cochlear cell	Fujioka et al. (2020)
**Amyotrophic lateral sclerosi**	Ropinirole hydrochloride	spinal motor neuron	Takahashi et al. (2019)
**Catecholaminergic polymorphicventricular tachycardia**	Dantrolene	cardiomyocyte	Penttinen et al. (2015)
**Friedreich ataxia**	Histone deacetylase inhibitor	Neuronal cell	Soragni et al. (2014)

**FIGURE 3:**
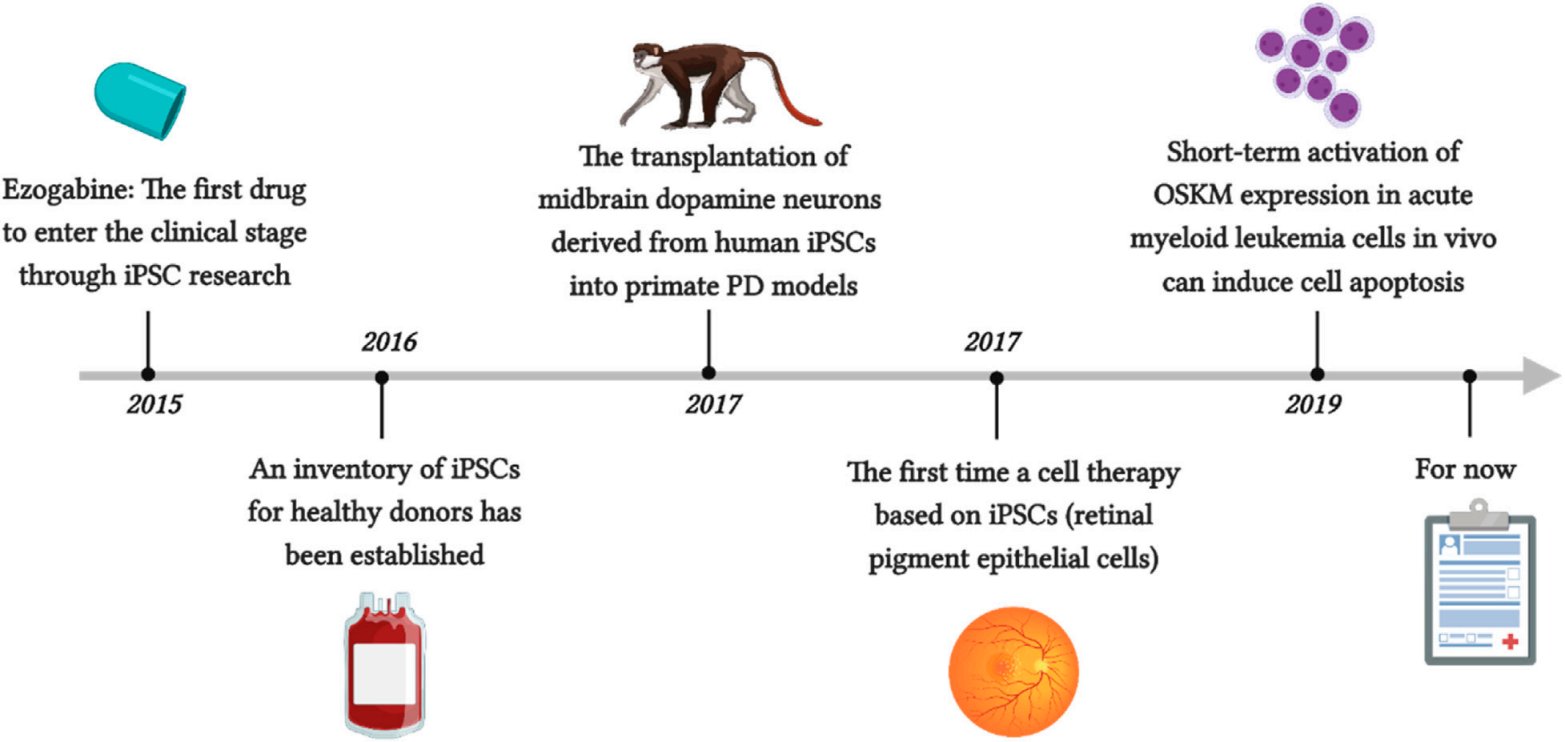
Representative disease modeling utilizing iPSCs.

McNeish reported the first drug to enter the clinical stage through iPSC research, ezogabine, which can regulate Kv7.2/3 class potassium channels through similar molecular mechanisms in patients with familial ALS (McNeish et al., 2015). For the first time, Mandai et al. reported a cell therapy based on iPSCs (Mandai et al., 2017). They prepared retinal pigment epithelial cells using autologous iPSCs made from the patient’s own fibroblasts and transplanted them as thin slices under the retina without the use of immunosuppressants to treat neovascular age-related macular degeneration (AMD). After 1 year, the patient’s vision also stabilized. Although the patient was not completely cured, the progression of his condition was slowed, which indirectly confirms the effectiveness and safety of this method. Unfortunately, this method not only has a high monetary cost but also requires a significant amount of time, as the production of iPSCs must undergo security audits before they can continue to differentiate. Another issue is immune rejection. Although allogeneic iPSCs can be used, autologous pluripotent stem cells are inevitably the safest. Therefore, an inventory of iPSCs from healthy donors has been established to address this issue. The raw material is blood from donors who are homozygous for human leukocyte antigen (HLA), as these cells are expected to minimize the risk of tissue rejection after transplantation (Azuma and Yamanaka, 2016). Kikuchi et al. showed that the transplantation of midbrain dopamine neurons derived from human iPSCs into primate PD models achieved good function within 2 years, achieved the expected results, and did not cause severe immune responses (Kikuchi et al., 2017). This is also considered the final threshold for clinical trial approval. Wang et al. reported that short-term activation of OSKM expression in acute myeloid leukemia cells *in vivo* can induce cell apoptosis, while its impact on normal hematopoietic stem cells and progenitor cells is negligible (Figure 3) (Wang et al., 2019d). Interestingly, several studies have shown that through iPSC technology, T cells can be reprogrammed to escape depletion, which seems to demonstrate the potential of this method in cancer immunotherapy (Nishimura et al., 2013; Vizcardo et al., 2013).

Many drugs and candidate drugs, such as cancer drugs, have achieved good results in animals but have had unexpected side effects on the human body and have not entered the market. The application of human pluripotent stem cells has potentially solved this problem, and reprogramming cancer cells into inducible cancer-initiating cells (iCICs) may be a way to address these issues (Li et al., 2017; Verusingam et al., 2017; Bindhya et al., 2021; Taguchi et al., 2021; Ahn et al., 2022; Canals et al., 2022; Chen et al., 2022). This approach not only provides rich and stable human samples but also greatly reduces the cost of early detection of human toxic side effects in drug development.

### Cell derivation

iPSCs were initially established from mouse fibroblasts because these cells are easy to process and proliferate vigorously. However, the establishment of primary human fibroblasts requires skin biopsy, and the process and requirements for establishment are also high. Therefore, more easily obtainable cell sources, such as gastric cells, liver cells, bone marrow cells, renal epithelial cells in urine, umbilical cord blood, amniotic membrane cells, neural stem cells, progenitor cells, and melanocytes, as well as some peripheral blood cells, T cells, B cells, hematopoietic stem cells, and fibroblasts, have gradually been identified (Figure 1) (Aoi et al., 2008; Eminli et al., 2008; Hanna et al., 2008; Maherali et al., 2008; Loh et al., 2009; Sun et al., 2009; Utikal et al., 2009; Cai et al., 2010; Seki et al., 2010; Zhou et al., 2011; Okita et al., 2013). According to recent research, all cells in the human body seem to have the potential to be induced into pluripotent stem cells, although their efficiency varies (Cui et al., 2022). Moreover, iPSCs have been established not only from mouse and human cells but also from various animals, such as chickens, fish, rabbits, monkeys, dogs, pigs, goats, horses, and cows (Wang J. et al., 2013; Gao et al., 2014; Ma et al., 2014; Song et al., 2014; Chu et al., 2015; Fuet and Pain, 2017; Yang et al., 2018; Pessôa et al., 2019a; Pessôa et al., 2019b; Setthawong et al., 2019; Jiang et al., 2020; Chandrasekaran et al., 2021; Mao et al., 2021; Botigelli et al., 2022). Moreover, iPSCs have been successfully extracted from the fibroblasts of several highly endangered species, such as drill and northern white rhinoceroses, which may provide guidance for the protection and recovery of these species (Ben-Nun et al., 2011; Saragusty et al., 2016). Overall, the genome, epigenome, and transcriptional variations of iPSC cell lines may lead to differences in cell behavior, which has indelible significance for clinical and biomedical applications of cells and provides new ideas for establishing and selecting optimal iPSC cloning methods.

## Perspectives

In recent decades, the rapid development of technology has led to a considerable increase in the understanding of iPSCs. iPSCs provide unique and rich resources for studying the development of pluripotent states and various cell types. These cells have a significant impact on the medical field, as they have regenerative potential, challenging our definition of cellular identity and providing new ideas for research on disease development. The experimental compounds that were once only used for testing in animal models can now be used in live human cells, which is expected to save considerable economic and time costs in drug development. Here, we propose some limitations of the reprogramming methods or future issues that need to be addressed. First, most of the current research on reprogramming has been conducted *in vitro* and in populations or subpopulations of reprogrammed cells. In the future, the mechanisms of *in vivo* reprogramming and how it functions at the single-cell level should be investigated. Second, due to the different properties and durations of reprogramming factors, it is difficult for different studies to compare them in parallel, and the molecular basis for cell- and organ-specific reprogramming sensitivity is still not fully clear. Finally, endogenous regulatory factors in the body can be manipulated to make the reprogramming process nontumorigenic. In the more than 10 years since the first human report of iPSCs was published, iPSCs have gradually begun to be applied in clinical practice, and we believe that more encouraging and exciting results will be achieved in this field in the future.
